# Auxenochlorella: the green algal reference we’ve been waiting for

**DOI:** 10.1093/plcell/koaf278

**Published:** 2025-11-21

**Authors:** Regina Mencia

**Affiliations:** Assistant Features Editor, The Plant Cell, American Society of Plant Biologists; Cátedra de Biología Celular y Molecular, Facultad de Bioquímica y Ciencias Biológicas, Universidad Nacional del Litoral, Santa Fe 3000, Argentina

In biology, we use reference organisms to study, test, and observe natural phenomena. The ideal reference is one whose behavior represents a broad range of related organisms, is easy to handle, is amenable to modern genome manipulation techniques, and is adaptable to laboratory conditions. There are excellent references for some species, such as our dear *Arabidopsis thaliana* for flowering plants, but others lack an ideal representative. This is the case for green algae, which hold tremendous potential for discovering fundamental molecular and cellular processes, as well as for future biotechnological applications. The most commonly used species for studying green algae is *Chlamydomonas reinhardtii*, in which key mechanisms related to photosynthesis and chloroplast metabolism have been elucidated (Salome and Merchant 2019). However, Chlamydomonas does not offer the power of homologous recombination for facile nuclear genome manipulation.

In recent work, Rory J. Craig and colleagues (Craig et al. 2024) describe a new reference for green algal research: Auxenochlorella UTEX 250-A. The authors produced a high-quality, telomere-to-telomere phased diploid genome assembly using high-coverage Pacific Biosciences (PacBio) HiFi and linked-read Omni-C datasets, covering the 12 chromosomes from 2 haplotypes with a final genome size of 44 mb, along with the corresponding plastome (84.6 kb) and mitogenome (54.0 kb). Interestingly, while 6 of the chromosome pairs exhibit 1-to-1 homology, the other 6 were highly rearranged between the 2 haplotypes, suggesting that UTEX 250-A may be an allodiploid hybrid derived from the 2 closely related species *Auxenochlorella protothecoides* and *Auxenochlorella symbiontica* (see Fig.). Furthermore, 2 chromosomes were found to be trisomic. The genomic complexity of UTEX 250-A underscores the biological significance of these chromosomal rearrangements in algae, which may play a key role in adaptation and evolutionary diversification. To date, such mechanisms have been studied primarily in yeast, highlighting the potential of Auxenochlorella to broaden our understanding of genome dynamics in photosynthetic eukaryotes.

**Figure.**
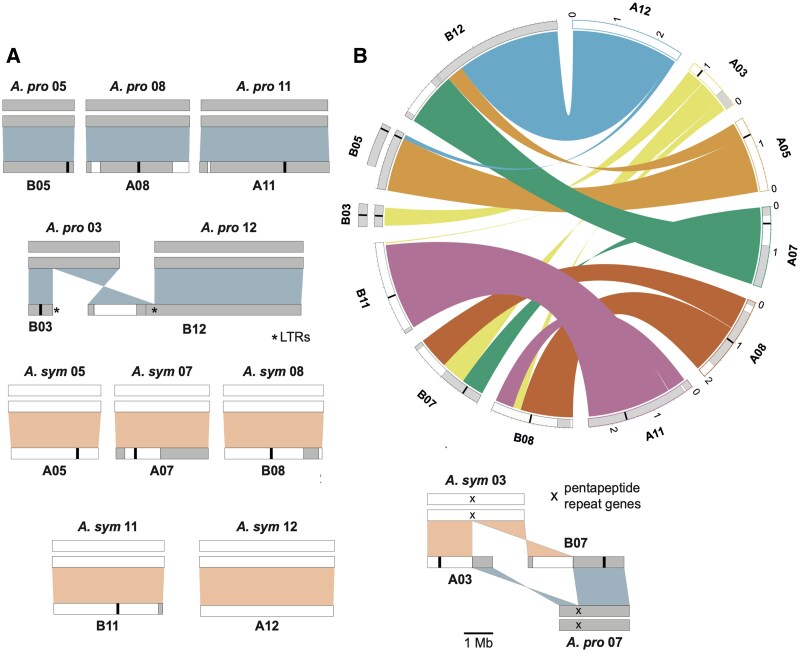
Allodiploid hybrid origin of Auxenochlorella UTEX 250-A. **A)** Comparison of the rearranged chromosomes of *UTEX 250-A* with those of the diploid species *A. protothecoides* and *A. symbiontica.*  **B)** Genomic rearrangements observed among 6 diploid *UTEX 250-A* chromosomes. Adapted from Craig et al. 2024, Figure 2.

Transcriptomic data from cultures grown under different conditions allowed functional annotation of genes, revealing several interesting findings, notably, the presence of multiple genes related to meiosis and sexual reproduction. Together with its hybrid origin, this raises the possibility that UTEX 250-A may be capable of sexual reproduction. Equally intriguing, the study identified extensive antisense long noncoding RNAs, mainly originating from bidirectional promoters, which may play regulatory roles in various cellular processes, particularly those related to DNA repair and recombination. In addition, several molecular tools were optimized, including selection markers such as Neomycin phosphotransferase II (nptII), which confers resistance to G418 (Geneticin); SUC2, which enables heterotrophic growth on sucrose; and MEL1, which allows heterotrophic growth on melibiose. To complement these tools, a set of fluorescent markers was designed for intracellular localization studies and labeling different organelles such as the cytoplasm, mitochondria, and plastids. To complete the characterization of UTEX 250-A as a proposed reference for green algae, the authors demonstrated that genomic manipulation through homologous recombination is achievable.

In conclusion, Auxenochlorella UTEX 250-A emerges as an attractive new reference for green algae, with unique features and advantages that could help address a wide range of research questions. The comprehensive work of Craig et al. (2024) provides detailed genome annotations and molecular tools that the plant research community can now build on and expand. Auxenochlorella provides a useful complement to Chlamydomonas within the diverse range of green algae. Because these genera diverged from a common ancestor ∼700 mYA, these 2 reference models together will improve our understanding of the breadth of biological processes within the Chlorophyta (see, e.g., Dueñas et al. 2025).

## Recent related articles in *The Plant Cell*:

- Dupuis et al. (2025) performed a systems-level study of *Chlamydomonas reinhardtii* under different light intensities, revealing that despite stable rhythmic gene expression, cells remodel photosystems and photoprotection as if “remembering” daily light cycles.
- Li et al. (2023) discovered unusually long (>26 nt) small RNAs in *Chlamydomonas reinhardtii* that bind AGO1 independently of Dicer, expanding under nutrient stress and hinting at an evolutionary link between transposon control and gene regulation.
- Peltier et al. (2024) quantified alternative photosynthetic electron flows in *Chlamydomonas reinhardtii*, showing that all can sustain CO₂ fixation but that chloroplast–mitochondrion coupling is the most energy-efficient.
- Zhao et al. (2023) solved the cryo-EM structure of the PSI–ACPI supercomplex in the cryptophyte *Chroomonas placoidea*, unveiling a transitional architecture that bridges red algal and diatom photosystems in the evolution of light harvesting.

## Dive Curated Terms

The following phenotypic, genotypic, and functional terms are of significance to the work described in this paper:

AGO1 Gramene: AT1G48410
AGO1 Araport: AT1G48410

SUC2 Gramene: AT1G22710
SUC2 Araport: AT1G22710

MEL1 Gramene: LOC_Os03g58600
MEL1 Araport: LOC_Os03g58600
